# B-type natriuretic peptide trumps other prognostic markers in patients assessed for coronary disease

**DOI:** 10.1186/s12916-019-1306-9

**Published:** 2019-04-03

**Authors:** Dipak Kotecha, Marcus D. Flather, Dan Atar, Peter Collins, John Pepper, Elizabeth Jenkins, Christopher M. Reid, David Eccleston, on behalf of the Alternative Risk Markers in Coronary Artery Disease (ARM-CAD) Study

**Affiliations:** 1 Institute of Cardiovascular Sciences, University of Birmingham, Birmingham, UK; 2 Monash Centre of Cardiovascular Research and Education in Therapeutics, School of Public Health and Preventive Medicine, Melbourne, Australia; 3 Faculty of Medicine and Health Sciences, University of East Anglia, Norwich, UK; 4 Department of Cardiology, Oslo University Hospital Ulleval, University of Oslo, Oslo, Norway; 5 Royal Brompton Hospital and National Heart and Lung Institute, Imperial College London, London, UK; 6 Baker Heart and Diabetes Institute, Melbourne, Australia; 7 Faculty of Health Sciences, School of Public Health, Curtin University, Perth, Australia

**Keywords:** Risk, Mortality, Coronary artery disease, Coronary angiography, B-type natriuretic peptide

## Abstract

**Background:**

Risk prediction for patients with suspected coronary artery disease is complex due to the common occurrence of prior cardiovascular disease and extensive risk modification in primary care. Numerous markers have the potential to predict prognosis and guide management, but we currently lack robust ‘real-world’ evidence for their use.

**Methods:**

Prospective, multicentre observational study of consecutive patients referred for elective coronary angiography. Clinicians were blinded to all risk assessments, consisting of conventional factors, radial artery pulse wave analysis, 5-minute heart rate variability, high-sensitivity C-reactive protein and B-type natriuretic peptide (BNP). Blinded, independent adjudication was performed for all-cause mortality and the composite of death, myocardial infarction or stroke, analysed with Cox proportional hazards regression.

**Results:**

Five hundred twenty-two patients were assessed with median age 66 years and 21% prior revascularization. Median baseline left ventricular ejection fraction was 64%, and 62% had ≥ 50% stenosis on angiography. During 5.0 years median follow-up, 30% underwent percutaneous and 16% surgical revascularization. In multivariate analysis, only age and BNP were independently associated with outcomes. The adjusted hazard ratio per log unit increase in BNP was 2.15 for mortality (95% CI 1.45–3.19; *p* = 0.0001) and 1.27 for composite events (1.04–1.54; *p* = 0.018). Patients with baseline BNP > 100 pg/mL had substantially higher mortality and composite events (20.9% and 32.2%) than those with BNP ≤ 100 pg/mL (5.6% and 15.5%). BNP improved both classification and discrimination of outcomes (*p* ≤ 0.003), regardless of left ventricular systolic function. Conversely, high-sensitivity C-reactive protein, pulse wave analysis and heart rate variability were unrelated to prognosis at 5 years after risk modification and treatment of coronary disease.

**Conclusions:**

Conventional risk factors and other markers of arterial compliance, inflammation and autonomic function have limited value for prediction of outcomes in risk-modified patients assessed for coronary disease. BNP can independently identify patients with subtle impairment of cardiac function that might benefit from more intensive management.

**Trial registration:**

Clinicaltrials.gov, NCT00403351 Registered on 22 November 2006

**Electronic supplementary material:**

The online version of this article (10.1186/s12916-019-1306-9) contains supplementary material, which is available to authorized users.

## Introduction

The prediction of adverse cardiovascular events and mortality is well described for patients without cardiovascular disease (CVD) [1, 2]. However, many patients have prior myocardial infarction (MI) or other CVD, invalidating standard risk scores such as Framingham. Further, patients have often received extensive primary and secondary prevention therapy (antiplatelet and antihypertensive therapy, lipid-lowering, smoking cessation and revascularization). Thus, conventional risk factors are often poorly associated with coronary artery disease (CAD) [3] or prognosis in those with established CVD [4, 5]. This leaves limited scope for identifying patients at high risk that might benefit from more intensive management.

A number of different risk markers with potentially novel mechanisms have been proposed to complement clinical factors. B-type natriuretic peptide (BNP) assesses cardiac strain and function [6], but is rarely considered in clinical practice outside of heart failure assessment despite suggestive evidence of value in CAD patients [7]. Pulse wave technologies are a surrogate for vascular stiffness [8]; however, their value beyond standard blood pressure is uncertain. Heart rate variability (HRV) is a marker of autonomic function [9], but with unknown effectiveness for risk prediction [10]. High-sensitivity C-reactive protein (hs-CRP) is an effective marker of inflammation, but additional risk stratification has not been established [11].

The Alternative Risk Markers in Coronary Artery Disease (ARM-CAD) study was designed to provide an unbiased assessment of non-invasive markers in a ‘real-world’ population. A randomised trial would not have been ethical in this situation, so instead we employed numerous methods to reduce potential bias. Risk assessment was performed prior to angiography, clinicians were blinded to results to avoid any impact on treatment over the 5 years of follow-up, and outcomes were independently adjudicated. Our aim was to establish the clinical value of these risk markers by assessing their relationship with mortality and the composite of death, MI or stroke, both early and late after planned coronary angiography.

## Methods

### Patient population

Patients referred for elective coronary angiography were recruited in three centres in Melbourne, Australia, with consecutive enrolment 2006–2008 following written informed consent. The only exclusion criteria were a precipitating acute coronary syndrome (ACS) or prior heart transplantation. All patients were assessed prospectively, prior to angiography, with the cardiologists blinded to all risk assessments throughout the follow-up period. The study was approved by local ethics committees, conducted according to the Declaration of Helsinki, and prospectively registered (https://clinicaltrials.gov/ct2/show/NCT00403351).

### Risk markers

Conventional risk factors were determined at patient study visits and with blood testing, in addition to careful review and confirmation using electronic health records and medical notes. Information on participants was collated with a bespoke electronic case report system separate from any clinical databases. Definitions on risk markers have previously been published [3, 8, 9], and further detail is presented in Additional file 1: Appendix 1.

### Coronary angiography

Coronary angiography was performed by experienced operators using standardised procedures. Patients were classified as normal, minor plaque or the number of coronary artery territories with a luminal stenosis ≥ 50% in main vessels or major tributaries. To ensure consistency, the angiographic core laboratory randomly evaluated angiograms during the study and at each centre, with two experienced, blinded operators.

### Outcomes

All-cause mortality and the composite of death, MI or stroke were independently adjudicated by clinicians blinded to patient details. Confirmation of events required documentary evidence supporting the diagnosis (for example, a death certificate, cardiologist or neurologist diagnosis, or troponin-positive ACS). We also collected detailed information about percutaneous coronary intervention (PCI), coronary artery bypass grafting (CABG) and other events during follow-up by reviewing electronic and physical notes, discussion with the patients’ cardiologist or general practitioner, and yearly telephone interviews with participants.

### Statistics

Values are presented as median ± interquartile range (IQR; 25th to 75th centiles) or percentage. Risk factor variables were assessed by tertile for Kaplan-Meier analysis and continuously in Cox proportional hazard regression. Where variables demonstrated a skewed distribution (for example, BNP, hs-CRP, HRV power and risk scores), these were normalised for statistical analysis by taking the natural logarithm. We pre-specified cut-points for a number of variables of interest: BNP 100 pg/mL, central augmentation pressure 24 mmHg, central pulse pressure 50 mmHg, low-frequency HRV 250 ms^2^ and hs-CRP 3 mg/L. Group comparisons were assessed with the Kruskal-Wallis non-parametric analysis of variance test, with *p* value adjustment for multiple comparisons. Kaplan-Meier groups were compared with the log-rank test of equality for binary variables and a log-rank trend test for tertiles.

The main multivariate Cox model consisted of age, gender, current smoking, systolic and diastolic blood pressure, use of renin-angiotensin-aldosterone antagonists, total cholesterol, statin therapy, diabetes, prior MI, BNP, the extent of angiographic CAD, the presence of left ventricular systolic dysfunction and revascularization during follow-up. Additional risk markers were then added separately. A time-varying interaction term was also included, and results are presented as hazard ratios (HR) with associated 95% confidence intervals (CI). We also performed stepwise inclusion at a threshold of *p* < 0.2 of a much broader range of variables (including renal function, body mass index and other medical therapy); this had no appreciable effect on results. Interactions were assessed with likelihood ratio testing, and the proportional hazards assumption was confirmed using Schoenfeld residuals. To understand the impact of risk factor modification over time, analyses were also performed at a 1-year landmark time point.

Harrell’s C-statistic (the area under the receiver operator curve) and Somers’ D-statistic were calculated in the final model with and without BNP to assess its additive value. These statistics were compared using published methods [12], with randomly generated derivation and validation sets of equal size, stratified by age and left ventricular systolic dysfunction. To determine the discriminatory ability of baseline BNP, we calculated the net reclassification improvement and integrated discrimination improvement using logistic regression in a multivariate model adding BNP to conventional clinical risk predictors (age, male gender, family history of premature CVD, current smoking, prior MI, diabetes, blood pressure ≥ 140/≥ 90 mmHg or hypertensive therapy, total cholesterol > 5.2 mmol/L [200 mg/dL], ≥ 50% stenosis on angiography and left ventricular ejection fraction [LVEF] < 50%) [13]. To reflect the high event proportions, predicted risk of death cut-points were a priori selected at 20%, 30% and 40%.

Post hoc analyses were performed to (1) exclude patients with atrial arrhythmias from central augmentation pressure and HRV Kaplan-Meier plots, (2) assess the impact of HRV in patients without revascularization, (3) identify any interaction of BNP with the severity of CAD and diastolic dysfunction, (4) exclude patients with entirely normal coronary angiography or those with left ventricular systolic dysfunction, and (5) evaluate the pre-specified 100 pg/mL cut-point of BNP.

A two-tailed *p* value of < 0.05 was considered statistically significant. Analyses used complete case data as the amount of missing data was small (no imputation performed). Statistical analysis was performed with Stata (version 14.2, StataCorp LP, TX, USA).

## Results

**Fig. 1:**
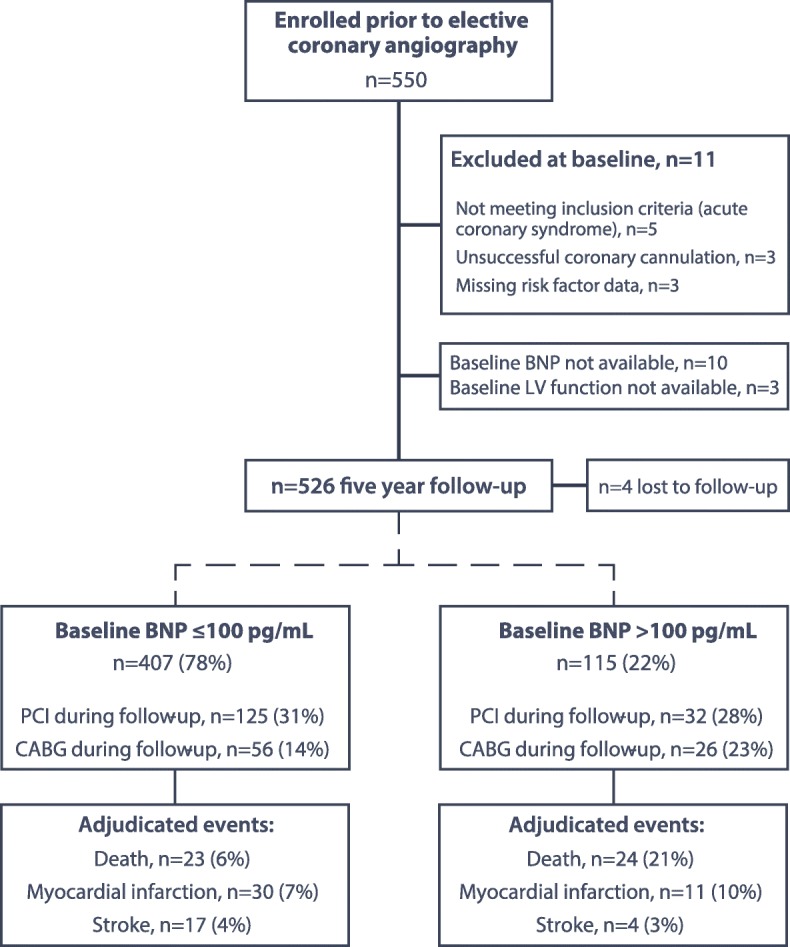
Flowchart for the ARM-CAD study. Includes independently adjudicated events subdivided by baseline BNP level. BNP, B-type natriuretic peptide; PCI, percutaneous coronary intervention; CABG, coronary artery bypass grafting (PCI and CABG are not mutually exclusive)

The ARM-CAD longitudinal study population consists of 526 patients recruited prior to elective coronary angiography, with a median follow-up period of 5.0 years (IQR 3.5–6.0). Follow-up data were unavailable in four participants (0.8%, Fig. 1).

### Baseline characteristics and associations

Table 1 reports the baseline demographics for the study population, with median age 66 years (IQR 58–73) and LVEF 64% (IQR 53–71%). Prior to angiography, 394 patients (75.5%) disclosed chest pain and 335 (64.2%) some degree of dyspnoea. Twelve patients (2.3%) had a history of a congestive heart failure episode. There was a broad range of BNP values which correlated with the degree of left ventricular systolic dysfunction, but not central pulse pressure (Additional file 1: Appendix 2). BNP was in the heart failure range (> 400 pg/mL) in only 5.6%.

**Table 1:** Baseline characteristics

Characteristic	All *N* = 522	BNP ≤ 100 pg/mL *N* = 407	BNP > 100 pg/mL *N* = 115	*p* value
	Median (IQR) or %	Median (IQR) or %	Median (IQR) or %	
Age, years	66 (58–73)	64 (56–71)	70 (65–76)	< 0.0001
Women, %	32.8%	33.4%	30.4%	NS
Current smoker, %	16.1%	17.2%	12.2%	NS
Prior myocardial infarction, %	22.2%	20.9%	27.0%	NS
Prior revascularization, %	20.9%	19.9%	24.3%	NS
Diabetes mellitus, %	22.0%	23.3%	17.4%	NS
Left ventricular ejection fraction, %*	64 (53–71)	65 (58–72)	53 (39–67)	< 0.0001
Left ventricular systolic dysfunction, %	19.0%	13.0%	40.0%	< 0.0001
Body mass index, kg/m^2^	28 (25–31)	28 (26–31)	27 (25–31)	NS
Systolic blood pressure, mmHg	140 (129–156)	139 (129–153)	145 (131–161)	0.019
Diastolic blood pressure, mmHg	79 (72–86)	79 (73–86)	77 (69–85)	NS
Central augmentation pressure, mmHg^†^	16 (9–23)	15 (9–22)	20 (10–27)	0.003
≥ 24 mmHg	23.5%	18.7%	40.7%	0.0002
Central pulse pressure, mmHg^†^	50 (39–63)	49 (39–61)	56 (42–73)	0.001
≥ 50 mmHg	52.1%	49.1%	62.8%	0.13
Total cholesterol, mmol/L	4.5 (3.8–5.2)	4.5 (3.9–5.4)	4.1 (3.5–4.7)	< 0.0001
High-density lipoprotein cholesterol, mmol/L	1.2 (1.0–1.4)	1.2 (1.0–1.4)	1.2 (1.0–1.5)	NS
Estimated glomerular filtration rate, mL/min	82 (68–97)	85 (69–98)	78 (60–93)	0.002
Low-frequency heart rate variability, ms^2 ‡^	211 (72–470)	213 (85–446)	191 (27–1017)	NS
> 250 ms^2^	56.5%	57.4%	52.7%	NS
Total power heart rate variability, ms^2 ‡^	830 (323–1954)	766 (360–1764)	1101 (198–3150)	NS
High-sensitivity C-reactive protein, mg/L^⁋^	1.9 (0.9–4.0)	1.9 (0.8–3.9)	2.1 (1.0–4.5)	NS
> 3 mg/L	32.4%	31.9%	34.2%	NS
B-type natriuretic peptide, pg/mL	40 (15–90)	27 (12–53)	188 (137–413)	–
> 100 pg/mL	22.0%			
> 400 pg/mL	5.6%			
Framingham 10-year risk, %	11 (8–20)	11 (7–20)	13 (8–20)	NS
SCORE 10-year risk, %	9 (4–17)	8 (3–16)	12 (7–22)	< 0.0001

*NS* not significant (adjusted for multiple comparisons)

*Based on the subset of patients with echocardiography prior to angiography (*n* = 295)

^†^*n* = 8 missing

^‡^Participants with a stable ECG signal over 5 min (*n* = 464)

^⁋^*n* = 1 missing

### Coronary disease, revascularization and risk reduction

The majority of patients (80%) had some degree of atheroma on angiography at baseline and 62% had one or more luminal stenoses ≥ 50%. Over the 5 years of follow-up, 15.6% underwent CABG and 29.9% of participants had at least one PCI procedure (drug-eluting stents used in 55%). There were similar total numbers of revascularization procedures in those with BNP ≤ 100 pg/mL at baseline and > 100 pg/mL (*p* = 0.69; see Fig. 1 for breakdown and Additional file 1: Appendix 3 for Kaplan-Meier curves). Patients were actively treated with medical therapy even prior to angiography (Additional file 1: Appendix 4) by highly involved secondary care physicians—the mean number of hospital visits per person was 1.3 in the first 12 months following angiography (standard deviation 0.6 visits).

### Adjudicated outcomes

**Fig. 2:**
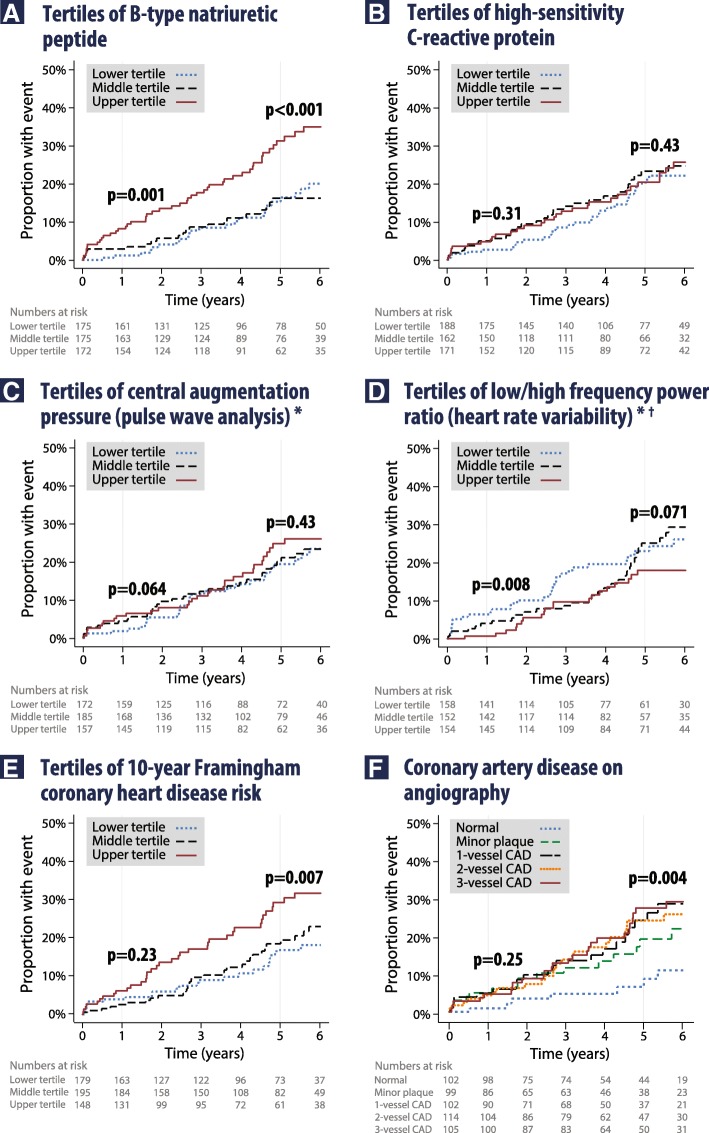
Kaplan Meier curves for death, myocardial infarction or stroke. Apart from coronary angiogram results, clinicians remained blinded to all other baseline risk markers. *p* values are a chi-squared log-rank test for trend performed at a landmark censoring of 1 year and at the median 5-year follow-up. Corresponding *p* values for all-cause mortality alone at 5-year follow-up are **a** BNP *p* = 0.001, **b** hs-CRP *p* = 0.27, **c** central augmentation pressure *p* = 0.38, **d** low/high-frequency HRV *p* = 0.30, **e** Framingham risk *p* = 0.026, **f** Angiographic coronary disease *p* = 0.09. *Post hoc exclusion of patients with atrial arrhythmias at baseline or follow-up had no impact on results. †Post hoc exclusion of patients with any revascularization resulted in *p*_trend_ = 0.029 for death, MI or CVA at 5-year follow-up, and 0.18 for mortality alone. CAD, coronary artery disease

Forty-seven participants (9.0%) died during follow-up. Cardiovascular causes accounted for 23 deaths (49%), of which 18 (38%) were due to cardiac causes and 5 (11%) from stroke. Adjudicated death, MI or stroke occurred in 100 participants (19.2%; 109 total events). Kaplan-Meier univariate analysis confirmed a significant trend across tertiles of BNP both at the 1-year landmark point and at median 5 years of follow-up (Fig. 2a). Hs-CRP (Fig. 2b) and central augmentation pressure (Fig. 2c) were unrelated to outcomes at either time point, whereas the ratio of low/high-frequency HRV power was significant at 1 year but not at 5 years (Fig. 2d). Conversely, both Framingham risk (Fig. 2e) and the extent of CAD on angiography (Fig. 2f) were non-significant at 1 year, but were statistically associated with adverse outcomes by the end of follow-up. Neither Framingham nor SCORE had value beyond age alone (Additional file 1: Appendix 5).

Across all multivariate models, the only individual baseline risk markers associated with adverse outcomes were age and BNP (Table 2 and Additional file 1: Appendix 6). For all-cause mortality, the HR for age per 10-year increment was 2.29 (95% CI 1.51–3.48; *p* = 0.0001) after adjustment for conventional risk factors, medical therapy, the extent of angiographic CAD and overt left ventricular systolic dysfunction. Per log unit increase in BNP, the adjusted HR was 2.15 (95% CI 1.45–3.19; *p* = 0.0001), with no interaction with the presence of left ventricular systolic dysfunction (*p*_interaction_ = 0.69). Similar findings were observed for death, MI or stroke. Both the Framingham and SCORE composite risk tools were associated with adverse outcomes in multivariate analysis, with no interaction according to baseline cardiovascular disease (all *p*_interactions_ ≥ 0.50; Table 2). A revascularization procedure during follow-up was associated with a significant reduction in the risk of death, with an adjusted HR of 0.45 (95% CI 0.23–0.87; *p* = 0.018).

**Table 2:** Multivariate analysis

	All-cause mortality			Death, MI or stroke		
	HR	95% CI	*p* value	HR	95% CI	*p* value
Main multivariate model*						
Age (per 10 years)	2.29	1.51–3.48	< 0.001	1.63	1.24–2.13	< 0.001
Male gender	1.82	0.86–3.82	0.12	1.91	1.10–3.33	0.022
Prior myocardial infarction	1.71	0.85–3.44	0.13	1.60	0.98–2.61	0.06
Diabetes	1.13	0.55–2.32	0.74	1.34	0.82–2.18	0.24
Smoking	1.39	0.63–3.10	0.41	1.39	0.78–2.47	0.26
Presence of impaired left ventricular function	1.32	0.63–2.77	0.46	1.22	0.72–2.08	0.46
Extent of angiographic CAD (per vessel with ≥ 50%)	1.03	0.77–1.39	0.84	0.90	0.72–1.13	0.38
Revascularization	0.45	0.23–0.87	0.018	1.18	0.73–1.91	0.49
Cholesterol (per 1 mmol/L)	1.09	0.79–1.49	0.60	1.14	0.91–1.42	0.26
Systolic blood pressure (per 1 mmHg)	0.99	0.98–1.01	0.36	0.99	0.98–1.01	0.36
BNP (per log unit)	2.15	1.45–3.19	< 0.001	1.27	1.04–1.54	0.018
Additional risk markers^†^						
Pulse wave analysis						
Central augmentation pressure (per 1 mmHg)	0.97	0.93–1.02	0.27	1.00	0.97–1.04	0.92
Central augmentation index (per 1%)	0.98	0.95–1.01	0.26	1.00	0.98–1.02	0.89
Central pulse pressure (per 1 mmHg)	0.98	0.93–1.04	0.59	1.00	0.96–1.04	0.88
Heart rate variability						
Standard deviation of RR intervals (per log ms)	0.88	0.57–1.37	0.58	0.91	0.67–1.26	0.58
Low-frequency power (per log ms^2^)	0.97	0.81–1.15	0.70	0.97	0.85–1.10	0.59
Total frequency power (per log ms^2^)	0.95	0.78–1.15	0.58	0.95	0.82–1.09	0.45
Low to high-frequency power ratio (per log unit)	1.00	0.73–1.38	0.99	0.96	0.78–1.18	0.72
Other biomarkers						
High-sensitivity C-reactive protein (per 1 mg/L)	1.11	0.85–1.46	0.43	1.00	0.84–1.20	0.98
Estimated glomerular filtration rate (per 10 mL/min)	0.94	0.81–1.09	0.40	0.99	0.90–1.08	0.76
Conventional risk scores^‡^						
Framingham 10-year risk (per log unit) ^⁋^	1.69	1.19–2.41	0.004	1.64	1.00–2.68	0.049
SCORE 10-year risk (per log unit)^⁑^	1.41	1.14–1.74	0.002	1.59	1.16–2.17	0.004

*Also adjusted for statin therapy, diastolic blood pressure and use of renin-angiotensin-aldosterone antagonists

^†^Variables separately added to the main multivariate model. Age and BNP remained significantly associated with outcomes in all models

^‡^Modified model that includes the risk score, prior MI, extent of angiographic CAD, impaired left ventricular function, BNP, use of renin-angiotensin-aldosterone antagonists, use of statin therapy and revascularization on follow-up, but not the components of the risk score

^⁋^*p*_interaction_ for baseline cardiovascular disease = 0.76 for all-cause mortality and 0.50 for death, MI or stroke

^⁑^*p*_interaction_ for baseline cardiovascular disease = 0.96 for all-cause mortality and 0.74 for death, MI or stroke

**Fig. 3:**
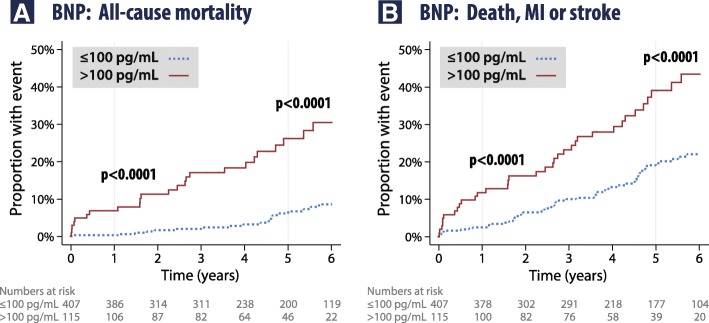
Kaplan-Meier adverse event curves according to the pre-specified BNP cut-point of 100 pg/mL for **a**: all-cause mortality; and **b**: death, MI or stroke. BNP, B-type natriuretic peptide; MI, myocardial infarction

Patients with baseline BNP > 100 pg/mL had substantially more adverse outcomes. Deaths occurred in 24/115 (20.9%) versus 23/407 (5.7%) in patients with BNP ≤ 100 pg/mL, with an adjusted HR of 4.49 (95% CI 2.09–9.62; *p* = 0.0001) and no interactions in subgroup analysis (Additional file 1: Appendix 6). Death, MI or stroke occurred in 37/115 (32.2%) with BNP > 100 pg/mL versus 63/407 (15.5%) with BNP ≤ 100 pg/mL, with an adjusted HR of 1.95 (95% CI 1.20–3.18; *p* = 0.007). The Kaplan-Meier survival curves are presented in Fig. 3, with clear and early separation in patients with baseline BNP > 100 pg/mL for both outcomes. No other cut-points were of statistical relevance, including those for central augmentation pressure, central pulse pressure, low-frequency HRV power and hs-CRP (Additional file 1: Appendix 7). In sensitivity analyses, BNP > 100 pg/mL had the same association with composite outcomes after exclusion of patients with normal coronary angiography (adjusted HR 2.03, 95% CI 1.23–3.34; *p* = 0.006; *n* = 420) or exclusion of those with any degree of left ventricular systolic dysfunction (adjusted HR 2.55, 95% CI 1.40–4.61; *p* = 0.002; *n* = 423).

### Discrimination value of BNP

**Fig. 4:**
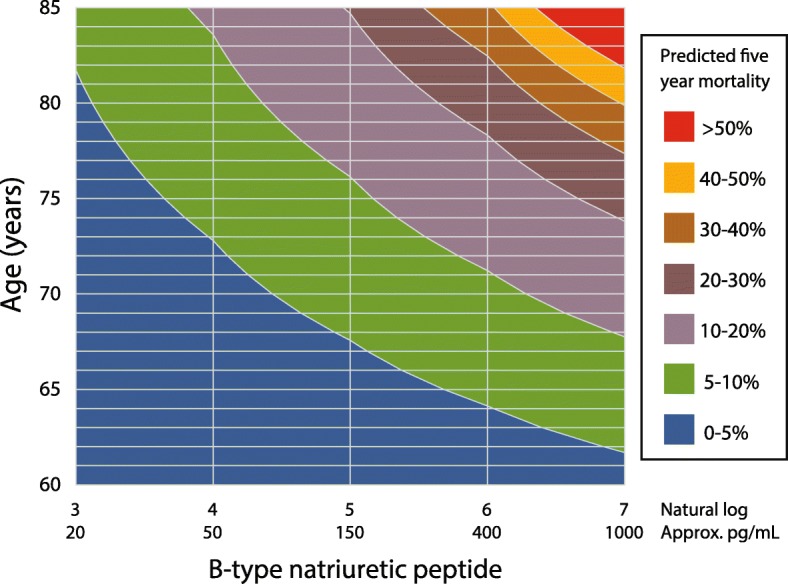
Risk contour map for adjusted predicted mortality according to age and BNP. Example: 75-year-old patient with a BNP of 25 pg/mL has a predicted 5-year mortality of 0–5%, compared to 10–20% in a patient of the same age with a BNP of 200 pg/mL. Note that risk estimates are based on the ARM-CAD cohort of patients with extensive modification of risk factors and concomitant disease, revascularization as required, and highly involved secondary care physicians

The addition of BNP as a continuous variable increased both Harrell’s C and Somers’ D statistics in multivariate models (*t* = 4.0, *p* < 0.001 and *t* = 5.9, *p* < 0.001 respectively for all-cause mortality). The C-statistic in the random validation subset was 0.91 with BNP and 0.69 without. When added to conventional risk predictors, BNP > 100 versus ≤ 100 pg/mL improved the classification of death (net improvement in 10 cases from 47 deaths, 21.3%), with minimal change in the classification of survivors (net improvement in 2 cases from 475 survivors, 0.4%; Additional file 1: Appendix 8). Both net reclassification improvement (NRI) and integrated discrimination improvement (IDI) analyses were highly significant (NRI 0.281, *p* = 0.003; IDI 0.038, *p* = 0.0007). Figure 4 displays a contour prediction map combining age and BNP to assess the clinical risk of 5-year mortality.

A plain English summary of results for patients and carers is presented in Additional file 2.

## Discussion

Our study extends the importance of BNP, a marker of myocardial dysfunction, to include adverse cardiovascular events and death in patients with suspected CAD, even at low elevation of BNP (suggestive of subtle cardiac impairment). The association of BNP with prognosis was independent of overt left ventricular systolic dysfunction, age and other risk factors and persisted despite effective primary and secondary risk factor modification and high rates of revascularisation over 5 years. On the contrary, other risk markers that focus on vascular pressure and arterial compliance (PWA), inflammatory responses (hs-CRP) and autonomic function (HRV) were not independent of risk modification. The strengths of the ARM-CAD study cohort were inclusion of unselected and consecutive patients (with results generalisable to routine clinical practice), blinding of risk assessment to clinicians, rigorous protocol-defined assessment and follow-up, low rates of loss to follow-up and independent adjudication of adverse outcomes.

### Cardiovascular risk assessment

Risk scores like GRACE (Global Registry of Acute Coronary Events) for ACS patients [14] confirm that many of the original Framingham Heart Study risk factors may lose relevance in patients with prevalent CVD. In our study, only age was independently associated with outcomes. This highlights the need to find alternative markers that can identify those patients at high residual risk despite risk factor management, or in those with existing disease.

### Pulse wave analysis

Following two large outcome studies of patients free of major CVD [15, 16], derived aortic waveforms are now frequently used in research studies as surrogate outcome measures. Our analysis in comorbid patients, with nearly four times the number of events as a prior study [17], found no relationship between the arterial waveform and clinical outcomes. This may have been due to treatment of underlying risk factors and use of vasoactive medications, which are known to affect central pressures [18].

### Heart rate variability

Low HRV is associated with an increased risk of incident events in those without CVD [19], can discriminate patients with a complicated course following MI [20, 21] and is a prognostic marker in heart failure [22]. Our prior results confirmed that reduction in HRV was an independent predictor of the presence and severity of angiographic CAD [9]. In the current analysis, we identified a significant relationship with crude events at the landmark 1-year point, but no association at final follow-up, nor in multivariate analysis. This may have been due to the lack of power (however in this case, the clinical value is likely to be small), that HRV is not entirely independent of conventional factors, or that revascularisation may play a role [10]. When we excluded patients with any prior revascularisation, we identified a statistically significant trend for tertiles of the ratio of low/high-frequency HRV power at 5 years. However, this should be considered hypothesis-generating due to the smaller sample size (*n* = 223) and post hoc assessment. It is also important to note that HRV acquisition was unsuccessful in 11% of our cohort, predominantly due to rhythm abnormalities [9].

### Biomarkers

hs-CRP has been extensively studied as a marker of inflammation, but has relatively poor ability to alter clinical management [11]. In our blinded assessment of a cohort with extensive risk management, we found no relationship with long-term outcomes.

In contrast, BNP was independent of other risk factors and was the only individual marker (aside from age) to be associated with clinical outcomes in multivariate analysis. BNP is produced by the myocardium in response to wall stress and acts to reduce venous return to the heart through actions on the vascular endothelium (smooth muscle relaxation and increased endothelial permeability), the kidneys (stimulation of diuresis and natriuresis) and suppression of reflex sympathetic activation [23, 24]. In patients with CAD or ACS, BNP is a powerful predictor of death and other clinical events [25–27], with analogous findings for NT-proBNP [28, 29]. Of interest, BNP is not associated with clinical events in healthy patients with normal echocardiograms and the absence of cardiovascular risk factors [30]. The mechanism for a rise in BNP in patients with coronary atherosclerosis but the absence of myocardial necrosis is presumably transient left ventricular systolic dysfunction, causing myocardial stress and activation of BNP gene transcription. This hypothesis is suggested by evidence that a larger ischemic burden, and hence a greater volume of myocardium affected, leads to a proportionally higher elevation in BNP [31]. In our study, the severity of CAD taking account of disturbance in usual coronary blood flow did correlate with BNP levels, albeit weakly (Additional file 1: Appendix 9). However, there was no interaction with the association of BNP with adverse clinical events. The impact of hibernating myocardium and the nature of the ischaemic insult itself are confounding issues, further complicated by unapparent or transient left ventricular systolic or diastolic dysfunction [32, 33]. Like other studies, we demonstrate that BNP is a much more sensitive marker than imaging parameters of systolic dysfunction [29, 34] and also establish that BNP is a risk marker independent of risk modification or incident revascularization. Our BNP cut-point of 100 pg/mL was pre-specified, but in post hoc analysis was demonstrated as a good inflexion point for an increasing risk of clinical events (Additional file 1: Appendix 10).

Our data would support the use of natriuretic peptides in the routine assessment of not only heart failure patients, but also those under investigation for suspected CAD, even without overt systolic dysfunction. BNP and NT-proBNP are readily available across the world, relatively cheap and can improve both classification and discrimination for longer-term outcomes. At the time of initial patient assessment, identification of patients with subtle elevation in natriuretic peptide levels (BNP > 100 pg/mL; roughly equivalent to NT-proBNP > 300 pg/mL) suggests a high-risk profile. In such patients, it would be reasonable to consider further pharmacological or interventional management, although further research is required to ascertain whether pro-active, intensive therapy would improve prognosis.

### Limitations

As this study was observational, there remain potential biases to consider, including selection for coronary angiography (particularly in patients with known CAD or previous revascularization that are more likely to be offered angiography). BNP levels after CABG are known to be dependent on a number of interacting and variable factors [35]; all analyses were therefore repeated in a cohort excluding prior CABG, and results were identical to those presented. It is also important to note that revascularisation rates were similar, regardless of baseline (blinded) BNP category (Additional file 1: Appendix 3). Although clinicians were blinded to the risk assessments, we cannot exclude the possibility that biomarkers could have been tested in clinical practice during the follow-up period. The lack of heart failure outcomes is a limitation of the study. Assessment of diastolic dysfunction was not a protocol requirement in this study. Post hoc analysis in patients with available data (*n* = 142) showed that mitral inflow E/A ratio > 2, tissue Doppler E/e′ ratio ≥ 13 or composite indices of diastolic impairment did not interact with the association of BNP with outcomes (*p*_interaction_ = 0.16 for death, *p*_interaction_ = 0.72 for composite events).

## Conclusions

In a real-world population, conventional risk factors and other markers of arterial compliance, inflammation and autonomic function had limited value for prediction of long-term outcomes in risk-modified patients assessed for coronary disease. BNP had a strong association with death and cardiovascular events, even without heart failure or overt ventricular dysfunction, and independent of revascularisation. BNP > 100 pg/mL can identify patients with subtle impairment of cardiac function that may conceptually benefit from early, targeted and individualised management to improve prognosis.

## Additional files

Additional file 1: Supplementary methods, tables and figures. (PDF 1127 kb)

Additional file 2: Plain language summary for patients. (PDF 19 kb)

## Abbreviations

ACS: Acute coronary syndrome
BNP: B-type natriuretic peptide
CABG: Coronary artery bypass grafting
CAD: Coronary artery disease
CI: Confidence intervals
CVD: Cardiovascular disease
HR: Hazard ratios
HRV: Heart rate variability
hs-CRP: High-sensitivity C-reactive protein
IQR: Interquartile range
LVEF: Left ventricular ejection fraction
MI: Myocardial infarction
PCI: Percutaneous coronary intervention
