# Supplementary material for: B-type natriuretic peptide trumps other prognostic markers in patients assessed for coronary disease
Source: BMC Med. 2019 Apr 3;17:72. doi: 10.1186/s12916-019-1306-9 (PMC6448253; doi:10.1186/s12916-019-1306-9)

B-type natriuretic peptide trumps other prognostic markers in patients assessed for coronary disease (Kotecha et al. 2019)

BMC Medicine

ADDITIONAL FILE 1

Appendix 1: Supplementary methods 2

Appendix 2: BNP associations with left ventricular systolic function and pulse pressure 5

Appendix 3: Revascularization during follow-up 6

Appendix 4: Medical therapy at baseline 7

Appendix 5: Comparison of Framingham, SCORE and age alone 8

Appendix 6: Forest plot of main analysis and subgroups for BNP 9

Appendix 7: Kaplan Meier plots for other pre-specified cut-points 10

Appendix 8: Reclassification of deaths with addition of BNP 11

Appendix 9: Severity of coronary artery disease 12

Appendix 10: Assessment of the BNP cut-point of 100 pg/mL 13

Dipak Kotecha, Marcus D Flather, Dan Atar, Peter Collins, John Pepper, Elizabeth Jenkins, Christopher M Reid and David Eccleston, on behalf of the ARM-CAD Study

Appendix 1: Supplementary methods

Risk markers:

Overall risk using conventional markers was estimated using the Framingham 10-year absolute event risk of total coronary disease (including angina, recognized and unrecognized MI and coronary deaths) and the SCORE 10-year high-risk fatal cardiovascular disease tool (adjusted for diabetes) [1, 2]. Blood pressure was determined from averaging two of three resting measurements with the patient in a sitting position using a validated oscillometric meter approved by the British Hypertension Society (Omron 705/T9P; Omron Healthcare, Japan). BNP was analysed at a core laboratory using a chemiluminescent microparticle immunoassay of EDTA plasma, with Abbott Architect instrumentation (coefficient of variation with this assay <5.3%). Hs-CRP was analysed using an immunoturbidimetric assay. Estimated glomerular filtration rate (GFR) was determined by the Modification of Diet in Renal Disease formula. Left-ventricular function was determined by ventriculography during cardiac catheterization, or on recent echocardiography where this was contraindicated. Radial artery pulse wave analysis was performed using a non-invasive Millar tonometer and transformed using a generalized transfer function to produce an aortic pulse waveform (SphygmoCor, version 8.0; Atcor Medical, Sydney). Central augmentation pressure, central augmentation index and central pulse pressure were derived and quality-controlled as previously described [3]. HRV was measured in 464 participants with a stable ECG signal over a mean capture time of 5.4 minutes (SD 0.5) [4]. RR intervals were quantified and deconstructed into component frequencies of low, high and total HRV power (SphygmoCor, version 8.0; Atcor Medical, Sydney).

Sample size:

A sample size of 500 patients and 77 events was estimated for the composite outcome based on a power of 90% and alpha of 0.05 to detect a 15% higher event rate in high-risk patients (control

event rate 15%), accounting for 20% of patients in the high-risk stratum and 10% loss to follow-up (log-rank test of survivor functions; Freedman method). Although adjudicated MI rates were lower than expected, mortality rates were higher than anticipated; hence the total number of events surpassed requirements and the calculated power of our analysis is >0.95 for both outcomes at five years.

Post-hoc analysis on CAD severity:

Severity of CAD was measured in terms of the Leaman score[5] (as modified by the SYNTAX group[6]), which weights luminal narrowing with the usual blood flow of that coronary vessel. The Leaman score was only calculated in patients without prior CABG, and by definition excludes those with normal coronary angiography (total patients analysed = 263). Baseline correlations with the Leaman score in the ARM-CAD cohort have previously been published [7]. Leaman scores were log-transformed and correlated with log-transformed BNP using the Spearman test. The association of the Leaman score with outcomes was examined in Kaplan Meier plots, categorising the Leaman score into tertiles with significance from a log-rank trend test. Adjusted Cox hazard regression was used to test the interaction of the Leaman score with the relationship between BNP and death, MI or stroke.

Post-hoc analysis on BNP cut-point:

Assessment of the validity of the pre-specified BNP cut-point of 100 pg/mL was performed using a restricted cubic spline analysis of log-transformed BNP and the log-odds of the composite of death, MI or stroke during follow-up.

References for supplementary methods:

1. Wilson PW, D'Agostino RB, Levy D, Belanger AM, Silbershatz H, Kannel WB. Prediction of coronary heart disease using risk factor categories. Circulation. 1998;97:1837-1847.

2. Conroy RM, Pyorala K, Fitzgerald AP, Sans S, Menotti A, De Backer G, et al. Estimation of ten-year risk of fatal cardiovascular disease in Europe: the SCORE project. Eur Heart J. 2003;24:987-1003.

3. Kotecha D, New G, Collins P, Eccleston D, Krum H, Pepper J, et al. Radial artery pulse wave analysis for non-invasive assessment of coronary artery disease. Int J Cardiol. 2013;167:917-924.

4. Kotecha D, New G, Flather MD, Eccleston D, Pepper J, Krum H. Five-minute heart rate variability can predict obstructive angiographic coronary disease. Heart. 2012;98:395-401.

5. Leaman DM, Brower RW, Meester GT, Serruys P, van den Brand M. Coronary artery atherosclerosis: severity of the disease, severity of angina pectoris and compromised left ventricular function. Circulation. 1981;63:285-299.

6. Sianos G, Morel M, Kappetein A, Morice M, Colombo A, Dawkins K, et al. The SYNTAX Score: an angiographic tool grading the complexity of coronary artery disease. EuroInterv. 2005;1:219-227

7. Kotecha D, Flather M, McGrady M, Pepper J, New G, Krum H, et al. Contemporary predictors of coronary artery disease in patients referred for angiography. Eur J Cardiovasc Prev Rehabil. 2010;17:280-288.

Appendix 2: BNP associations with left ventricular systolic function and pulse pressure

Panel A: Box and whisker plot demonstrating B-type natriuretic peptide (BNP) values according to the severity of left-ventricular systolic dysfunction. Central white line indicates the median.

Panel B: Scatter plot of the natural logarithm of BNP versus central pulse pressure using pulse wave analysis. Dashed line is the linear regression line adjusted for age with 95% confidence intervals. Regression coefficient 0.03 per 10mmHg, p=0.42.

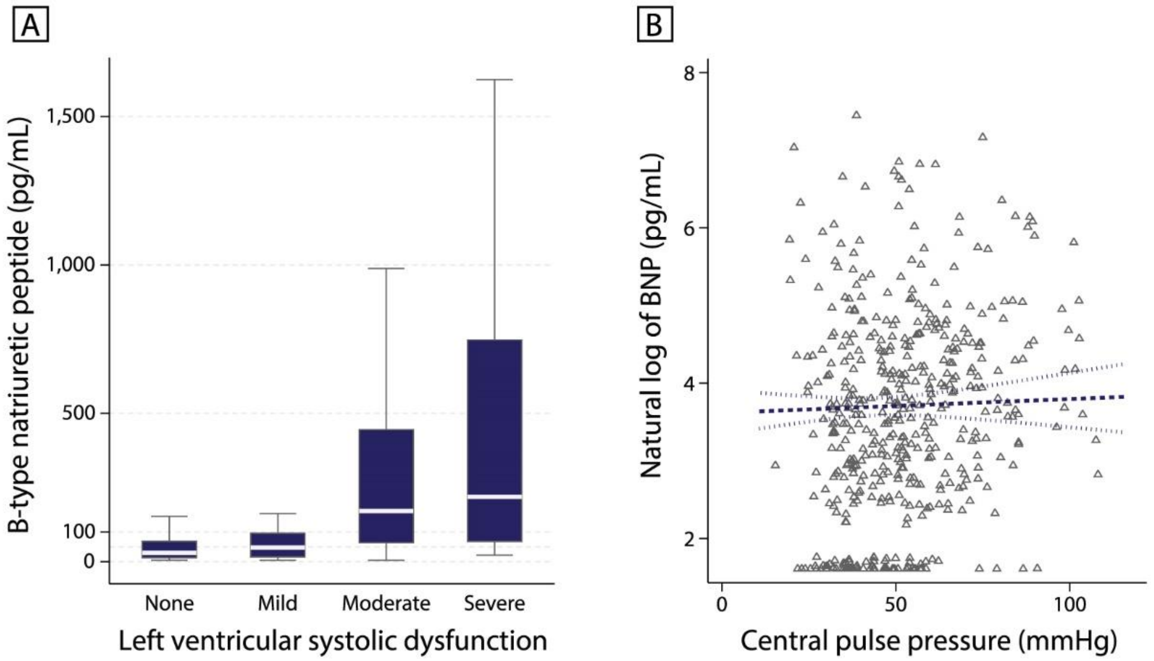

Appendix 3: Revascularization during follow-up

Kaplan Meier event curves for revascularization during follow-up according to baseline BNP level. Figure only includes patients with adjudicated event dates. BNP, B-type natriuretic peptide; CABG, coronary artery bypass grafting; PCI, percutaneous intervention.

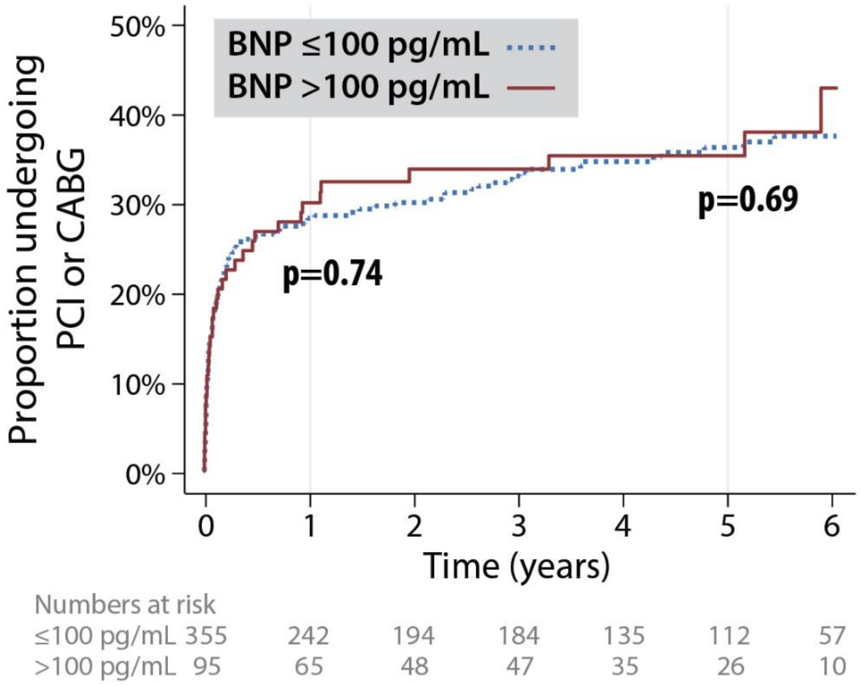

Appendix 4: Medical therapy at baseline

Baseline therapy	Percentage
Antiplatelet agent(s)	74.3%
Anticoagulant	5.4%
Renin-angiotensin-aldosterone antagonist	55.4%
Beta-blocker	45.2%
Calcium channel blocker	23.0%
Diuretic	19.0%
Oral vasodilator	18.0%
Statin	61.9%

Appendix 5: Comparison of Framingham, SCORE and age alone

Death, myocardial infarction or stroke Kaplan Meier event curves for tertiles of the Framingham and SCORE risk algorithms and baseline age alone.

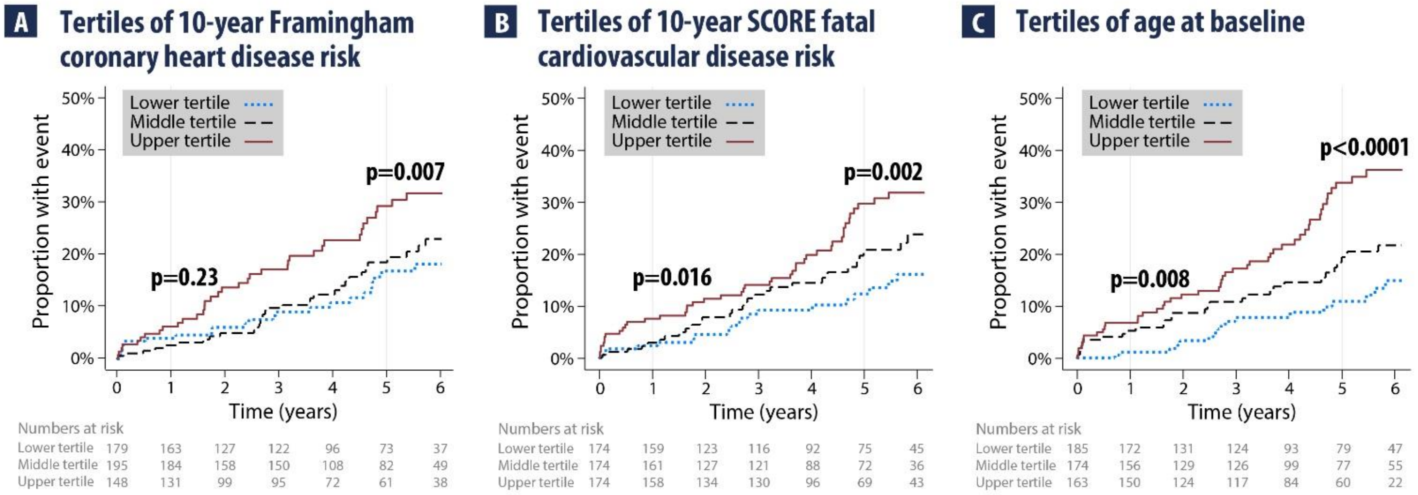

Appendix 6: Forest plot of main analysis and subgroups for BNP

Top section depicts multivariate-adjusted hazard ratios for major covariates (see Table 3 for other variables included in the Cox regression model). Lower section compares hazard ratios for BNP ≤100 versus >100 pg/mL according to patient subgroups.

BNP, B-type natriuretic peptide; CRP, C-reactive protein; LV, left-ventricular; RAAS, renin-angiotensin-aldosterone antagonists.

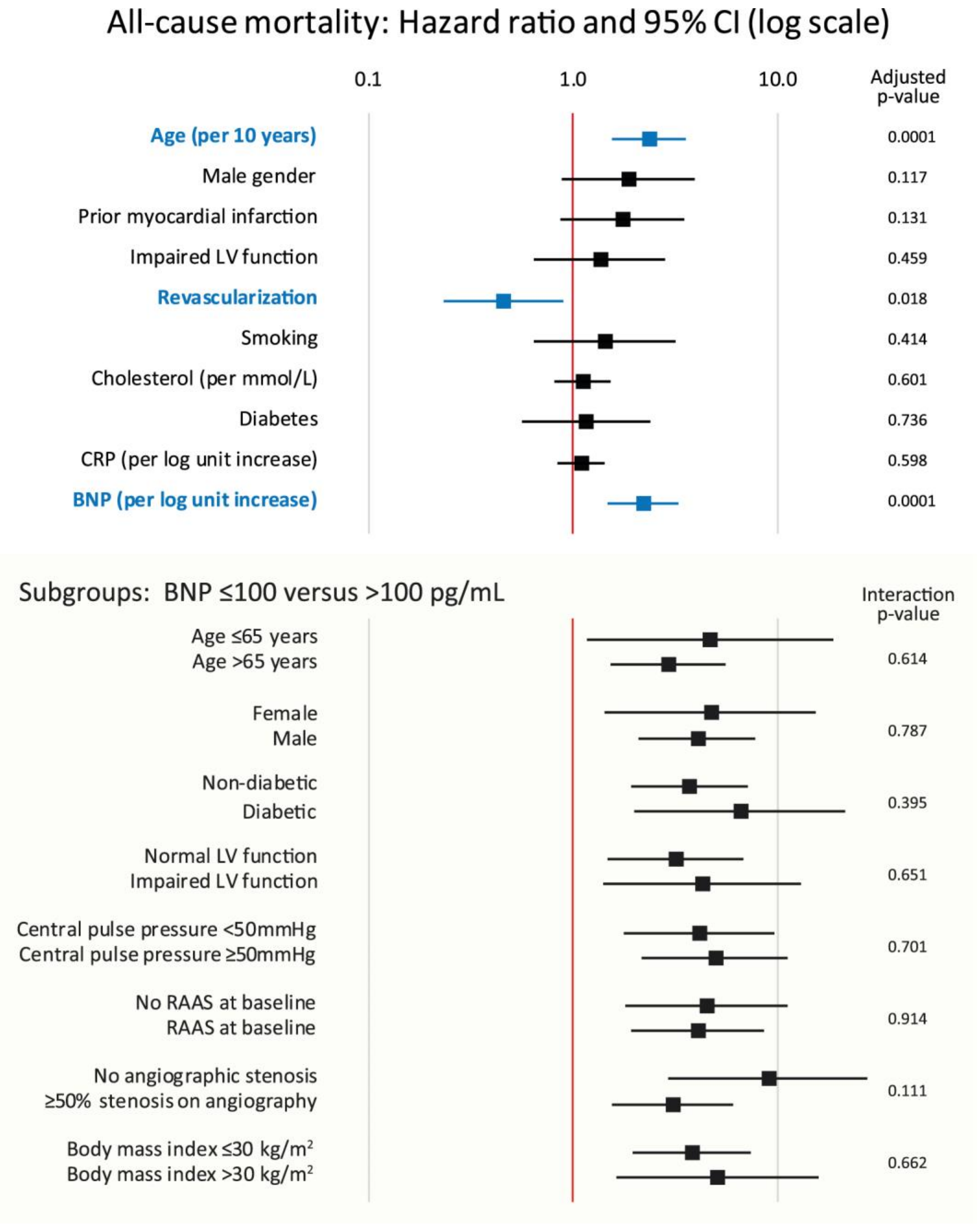

Appendix 7: Kaplan Meier plots for other pre-specified cut-points

Outcome of death, myocardial infarction or stroke. P-values are Chi-squared log-rank tests performed at a landmark censoring of 1-year and at the median 5-year follow-up. Corresponding p-values for all-cause mortality alone at 5-year follow-up are: (A) p=0.72; (B) p=0.95; (C) p=0.44; (D) p=0.31.

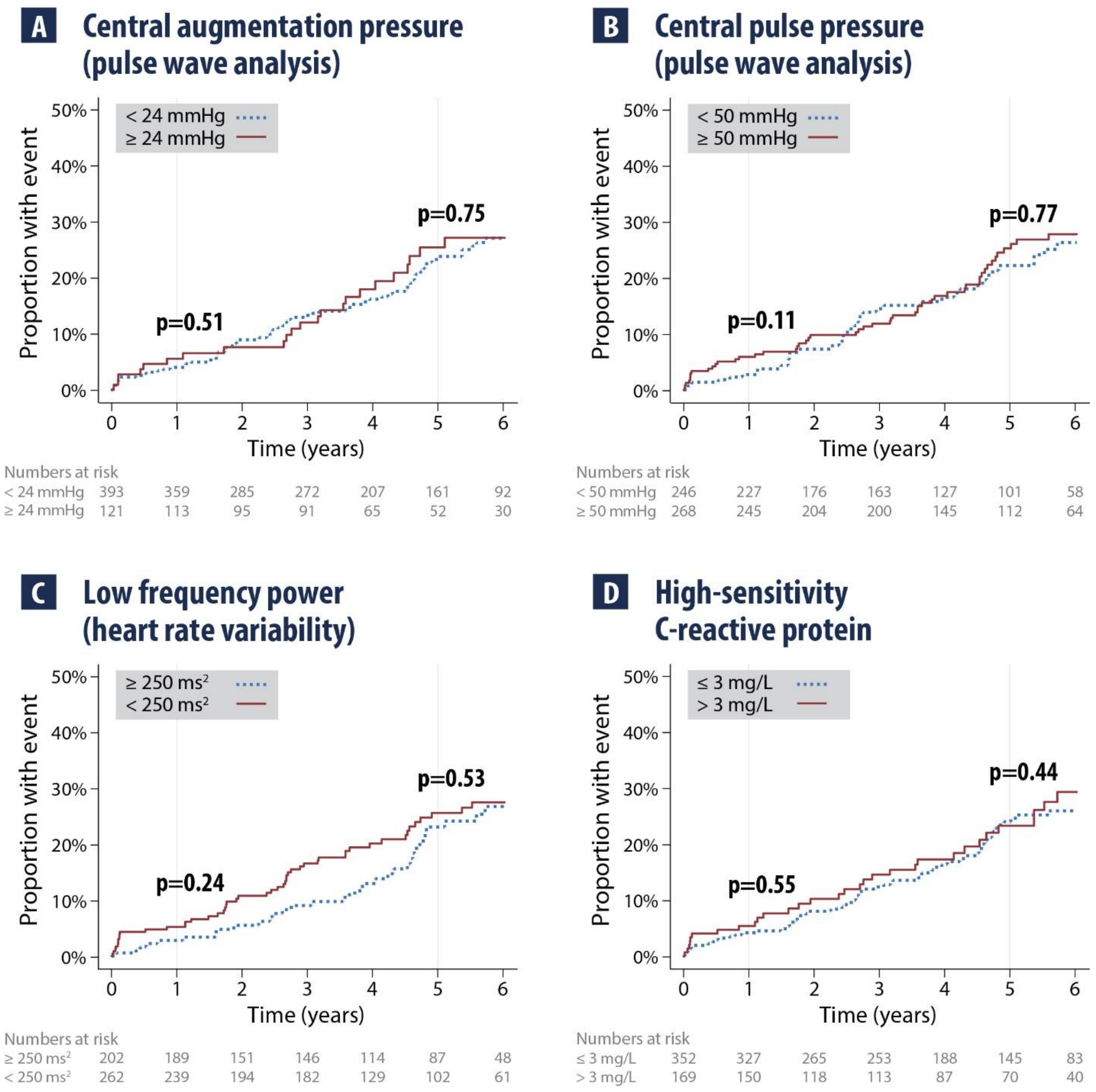

Appendix 8: Reclassification of deaths with addition of BNP

Risk prediction for mortality using conventional clinical risk predictors with and without B-type natriuretic peptide (>100 versus ≤100 pg/mL).

	Conventional risk factors only \ Conventional risk factors + BNP	<20%	20-30%	30-40%	≥40%	Total
**Died**	<20%	25	7	3		35
	20-30%	1		2	1	4
	30 - 40%		2			5
	≥40%				3	3
	Total	26	9	5	7	47
**Survived**	<20%	410	19	1		430
	20-30%	25	3	7	1	36
	30 - 40%	1	4		1	6
	≥40%			1	2	3
	Total	436	26	9	4	475

Key:	Positive impact	No change	Negative impact

Appendix 9: Severity of coronary artery disease

Correlation of Leaman CAD severity score with BNP:

Spearman correlation coefficient 0.24; p=0.001.

Kaplan Meier plot for association of Leaman CAD severity score with outcomes:

Outcome of death, myocardial infarction or stroke. P-values are Chi-squared log-rank tests performed at a landmark censoring of 1-year and at the median 5-year follow-up.

CAD, coronary artery disease.

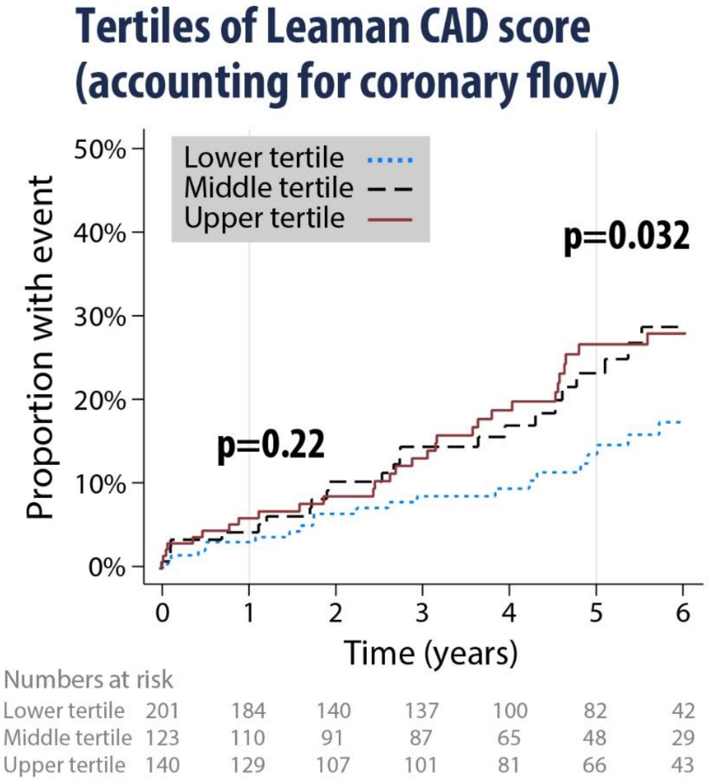

Interaction of Leaman CAD severity score and BNP:

Fully adjusted Cox hazard model for death, myocardial infarction or stroke;

p for interaction = 0.42.

Appendix 10: Assessment of the BNP cut-point of 100 pg/mL

Restricted cubic spline model, with BNP 100 pg/mL as the reference point for the odds of the composite outcome during the follow-up period.

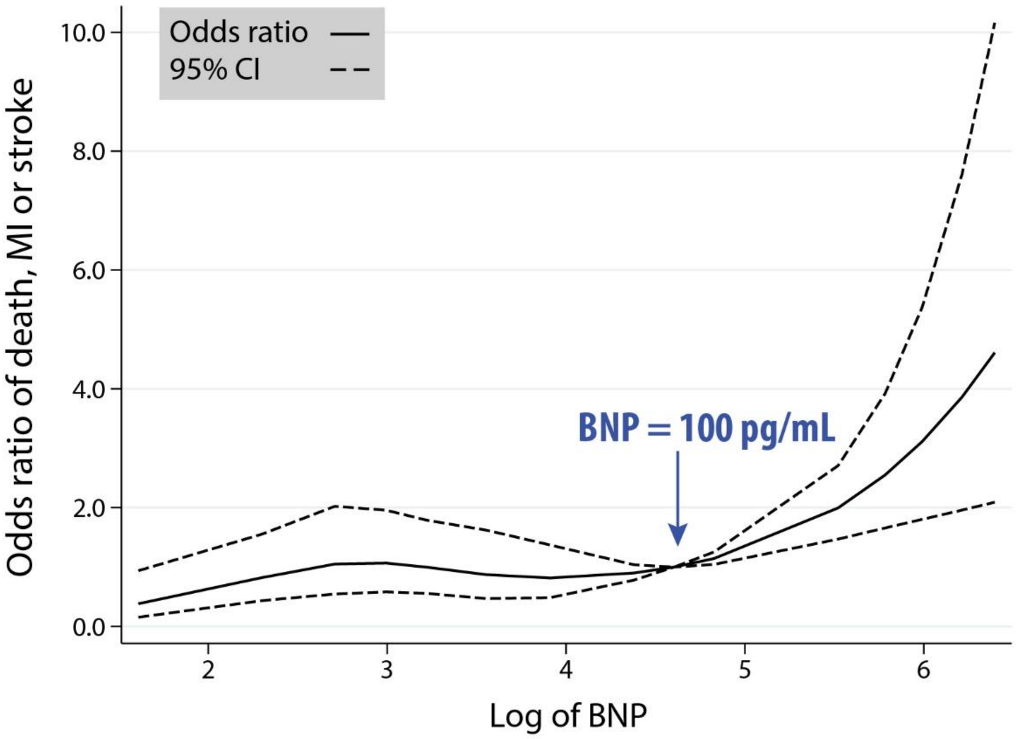

Supplement: Supplementary file 1 — Supplementary methods, tables and figures. (PDF 1127 kb) [file 12916_2019_1306_MOESM1_ESM.pdf]
