# Supplementary material for: B-type natriuretic peptide trumps other prognostic markers in patients assessed for coronary disease
Source: BMC Med. 2019 Apr 3;17:72. doi: 10.1186/s12916-019-1306-9 (PMC6448253; doi:10.1186/s12916-019-1306-9)
Supplement: Supplementary file 2 — Plain language summary for patients. (PDF 19 kb) [file 12916_2019_1306_MOESM2_ESM.pdf]

B-type natriuretic peptide trumps other prognostic markers in patients assessed for coronary disease (Kotecha et al. 2019)

BMC Medicine

ADDITIONAL FILE 2

PLAIN LANGUAGE SUMMARY FOR PATIENTS

It is often difficult to know which patients will suffer from heart attacks, strokes or could die early. Better assessment has the potential to lead to more targeted treatment by doctors. Most of the research in this area has focused on patients with no history of any previous heart condition. In this study we looked at all patients being considered for further investigation by a heart specialist, which includes those who already had disease of their heart or arteries, and those receiving treatments to reduce their risk of heart attacks.

We found that from a range of different types of risk factors, only older age and a blood marker called BNP were associated with death, heart attacks and strokes 5 years later. The BNP test is widely available around the world, and could be used to identify those patients who have a high chance of bad outcomes in the long-term. These patients could then receive more attention or different treatments to improve their well-being.

Dipak Kotecha, Marcus D Flather, Dan Atar, Peter Collins, John Pepper, Elizabeth Jenkins, Christopher M Reid and David Eccleston, on behalf of the ARM-CAD Study

ARM-CAD STUDY
